# Variations of Aberrant Volume, Activity, and Network Connectivity of Hippocampus in Adolescent Male Rats Exposed to Juvenile Stress

**DOI:** 10.3390/brainsci15030284

**Published:** 2025-03-07

**Authors:** Aoling Cai, Danhao Zheng, Fanyong Xu, Fei Wang, Sreedharan Sajikumar, Jie Wang

**Affiliations:** 1 Department of Radiology, Songjiang Hospital and Songjiang Research Institute, Shanghai Key Laboratory of Emotions and Affective Disorders, Shanghai Jiao Tong University School of Medicine, Shanghai 201600, China; aoling.wh@gmail.com (A.C.);; 2 Early Intervention Unit, Department of Psychiatry, Affiliated Nanjing Brain Hospital, Nanjing Medical University, Nanjing 210000, China; 3 Key Laboratory of Magnetic Resonance in Biological Systems, National Center for Magnetic Resonance in Wuhan, Wuhan Institute of Physics and Mathematics, Innovation Academy for Precision Measurement Science and Technology, Chinese Academy of Sciences, Wuhan 430071, China; 4 University of Chinese Academy of Sciences, Beijing 100049, China; 5 Department of Mental Health, School of Public Health, Nanjing Medical University, Nanjing 210000, China; 6 Department of Physiology, National University of Singapore, Singapore 117593, Singapore; 7 Life Sciences Institute Neurobiology Programme, Centre for Life Sciences, National University of Singapore, Singapore 117456, Singapore; 8 Healthy Longevity Translational Research Programme, Yong Loo Lin School of Medicine, National University of Singapore, Singapore 117456, Singapore

**Keywords:** juvenile stress, brain development, fMRI, brain volume, cognitive function

## Abstract

Background: Childhood is a crucial period for brain development, and short-term juvenile stress has demonstrated long-lasting effects on cognitive and cellular functions in the hippocampus. However, the influence of such stress on the brain’s overall network remains unclear. Methods: In this study, we employed functional magnetic resonance imaging (fMRI) to explore the effects of transient wild stress on juvenile male rats. Pregnant rats were purchased and housed in a specific pathogen-free (SPF) environment, with pups separated by sex on postnatal day 21 (PD21). From PD27 to PD29, male rats were subjected to transient wild stress, which included forced swimming, elevated platform exposure, and restraint stress. Following stress exposure, all animals were carefully maintained and scanned at 42 days of age (PD42) using fMRI. Structural analysis was performed using voxel-based morphometry (VBM) to assess changes in gray matter volume, while functional activity was evaluated through regional homogeneity (ReHo) and voxel-wise functional connectivity. Results: The results showed significant reductions in gray matter volume in several brain regions in the stress group, including the periaqueductal gray (PAG), entorhinal cortex (Ent), and dentate gyrus (DG). In terms of functional activity, cortical regions, particularly the primary somatosensory areas, exhibited decreased activity, whereas increased activity was observed in the PAG, DG, and medulla. Furthermore, functional connectivity analysis revealed a significant reduction in connectivity between the DG and entorhinal cortex, while the DG-PAG connectivity was significantly enhanced. Conclusions: These findings suggest that juvenile stress leads to profound alterations in both brain structure and function, potentially disrupting emotional regulation and memory processing by affecting the development and connectivity of key brain regions.

## 1. Introduction

Neuropsychiatric disorders often have their roots in early childhood, with their development closely linked to stressful life events during this critical period [1]. These adverse experiences not only have profound long-term impacts on adult life but also significantly increase the risk of mental health disorders [2]. Childhood is a pivotal stage for brain development, characterized by high synaptic plasticity, allowing environmental experiences to shape neural networks significantly [3]. However, this heightened plasticity also renders the brain particularly vulnerable to adverse experiences, potentially leading to lifelong behavioral abnormalities and neurofunctional disruptions.

Studies have demonstrated that juvenile stress (PD21-42) significantly impairs adolescent social behavior, manifesting as reduced social motivation, a decreased exploration of unfamiliar conspecifics, and a loss of preference for social novelty [4,5]. These behavioral traits closely resemble symptoms observed in patients with neuropsychiatric disorders, making them a critical model for investigating the neural mechanisms by which early-life stress affects social behavior [6]. However, the specific impact of early-life stress on the neural mechanisms regulating social behavior—particularly brain regions and networks associated with social memory—remains to be fully elucidated.

The hippocampus plays a critical role in processing social information and forming social memory, with the CA2 region emerging as a central contributor. Studies have shown that disrupting hippocampal function, such as the genetic silencing of CA2 neurons, results in deficits in the encoding, consolidation, and recall of social memory [7,8]. Notably, oxytocin receptors within CA2 are essential for distinguishing social stimuli, underscoring the region’s importance in social cognition [9]. Neural circuits linking the hippocampus, including connections from CA2 to ventral CA1 and the nucleus accumbens, form critical pathways for regulating social behavior and memory [10,11]. Alterations in hippocampal function, particularly in the CA2 region, have been implicated in neuropsychiatric disorders. For example, animal models of 22q11.2 microdeletion syndrome show age-related changes in CA2 neurons that parallel the progression of human diseases [12]. Environmental stress also significantly impacts hippocampal plasticity, with juvenile stress weakening CA2’s long-term plasticity by disrupting neuromodulatory inputs [13]. These findings highlight the hippocampus’s broader role in integrating social and environmental factors, shaping long-term social behaviors, and contributing to the risk of neuropsychiatric disorders.

While electrophysiological techniques excel in uncovering the functions of specific brain regions due to their high temporal resolution, their limited spatial coverage hinders the comprehensive exploration of whole-brain functional changes. Functional magnetic resonance imaging (fMRI), as a non-invasive imaging method, provides a unique advantage by quantifying dynamic variations in whole-brain activity, uncovering how early-life stress impacts the brain at a network level [14]. In particular, fMRI enables the analysis of functional connectivity between brain regions, offering insights into how stress disrupts neural network synchrony, altering overall brain functional states and behavioral outcomes.

Against this background, our study focuses on how juvenile stress affects the brain volume, regional activity and functional connectivity. While our analyses encompassed all brain regions without any prior assumptions, the results inevitably highlighted the hippocampus as a key region influenced by juvenile stress. By integrating measures of brain volume, regional activity, and network connectivity in an animal model, we illuminate the broad impacts of juvenile stress on brain structure, function, and connectivity from a whole-brain perspective.

## 2. Materials and Methods

### 2.1. Animal Preparation

The animals were housed in a specific pathogen-free (SPF) environment, with a temperature maintained between 20 °C and 25 °C. Food and water were provided with free access, consisting of a standard rodent maintenance diet and ultrapure water. The light cycle was set to 8:00 a.m. to 8:00 p.m. to ensure normal circadian rhythm. No interventions were applied outside of the experimental procedures, which began each morning. This experiment was conducted at the animal SPF conditions of the Institute of Precision Measurement and Technological Innovation

Pregnant Wistar rats were obtained from Beijing Vital River Laboratory. In total, eight pregnant rats were used in the study. Two pregnant rats were housed together until delivery. After birth, rat pups were weaned at postnatal day 21 (PD21) and separated into individual cages with 3–4 pups per cage. The male rats were randomly divided into two groups for further experiments, and female rats were excluded to avoid possible hormonal variations. The stress model was established after five days of habituation in the vivarium, and the MRI study was conducted when the rats reached 42 days of age. Animals from different groups (stress and control) were housed separately to avoid potential group effects.

### 2.2. Juvenile Stress

Juvenile stress (*n* = 11) exposure was conducted over three consecutive days (PD27~29, postnatal day) using the following procedures for the stress group. Stress exposures were carried out in a behavioral testing room with an ambient light intensity of approximately 100–200 lux.

- PD27 (forced swim stress): rats were placed in an opaque circular water tank (20 cm diameter, 45 cm height) filled with warm water (22 ± 2 °C) for 10 min.
- PD28 (elevated platform stress): rats were placed on a square transparent platform (21 × 21 cm) elevated 100 cm above the floor for 30 min in a confined room. This procedure was repeated three times, with a 60 min interval between the sessions.
- PD29 (restraint stress): rats were placed in a transparent acrylic box (11.5 × 5.5 × 4 cm) to restrict nearly all movement for 2 h under dim lighting conditions.

The control group (*n* = 15) remained undisturbed during the stress procedures, except for routine activities such as weighing and cage cleaning.

### 2.3. MRI Study

The MRI experiments were performed on a horizontal-bore 7.0 Tesla BioSpec MRI instrument (Bruker, Ettlingen, Germany), when the rats reached 42 days of age. A 72 mm birdcage transmit coil and a 10 mm surface coil was used in transmission and reception signals. Firstly, the animals were subjected to anesthesia by mixed air with 4% isoflurane (RWD Life Science Co., Shenzhen, China). They were then transferred onto an MRI animal bed and carefully fixed with ear bars and a bite bar. After that, a 10 mm surface coil was positioned right on the top of animal’s midbrain and a sustained dose of mixed air with 1% isoflurane was given to maintain the lightly anesthetized state. In addition, a warm water circulation system and a respiratory monitoring device were used to preserve and monitor the physiological state of animals.

The functional images were obtained using single-shot spin-echo planar imaging sequence (SE-EPI): repetition time (TR) = 2000 ms; echo time (TE) = 16 ms; field of view (FOV) = 24 × 24 mm^2^; matrix size = 64 × 64; slice thickness = 1 mm; gap = 0.1 mm; repetitions = 300. Anatomical images were obtained at the same geometry as the functional images using a fast spin-echo sequence (Turbo-RARE): TR = 2500 ms; TE = 12 ms; RARE factor = 8; FOV = 24 × 24 mm^2^; matrix = 256 × 256.

### 2.4. Voxel-Based Morphometry Analysis

T2-weighted images were initially co-registered to the template image using a rigid transformation (SPM, Coregister). Subsequently, segmentation was performed using the SPM old segment tool. All the probability density templates and the template images were obtained from the published SIGMA template (Version 1.1). After segmentation, the normalized T2-weighted images with voxel intensities modulated were smoothed using a Gaussian kernel with a full width at half maximum (FWHM) of [6 6 6]. The modulated, normalized gray matter images (mwc1) were then included in the final statistical analysis. The difference region between control and stress groups was shown by a two-sample *t*-test with GRF correction (voxel *p* < 0.05; cluster *p* < 0.05).

### 2.5. rs-fMRI Data Preprocessing

The data preprocessing was accomplished by spm12 (https://www.fil.ion.ucl.ac.uk, accessed on 2 January 2020), dpabiV3.1_180801 (http://www.rfmri.org, accessed on 1 January 2020), REST1.8 (http://restfmri.net, accessed on 1 January 2020) and in-house codes on Matlab_R2014a (Mathworks, Natick, MA, USA). Firstly, the data were converted to NIFTI format with 10-fold expansion on voxels size using MRI instrument assorted softwareBru2Nii (https://github.com/neurolabusc/Bru2Nii, accessed on 1 January 2020). After that, the data were corrected for slice timing and flipped to fit the orientation of template. For the statistical analysis of the groups, the fMRI data were normalized to a home-made EPI template. Furthermore, a series of processing programs was used to improve the data quality in the temporal dimension, such as detrend (linear), head motion regressor (Friston 24), filter (0.01~0.1 Hz), and scrubbing (FD Jenkinson < 0.2). Smooth (FWHM [6 6 6]) was optional, depending on the requirements of subsequent analyses.

### 2.6. Brain Regional Activity Analysis

This study employed regional homogeneity (ReHo) to assess local brain activity. ReHo quantifies the synchronization of neural activity by calculating the consistency of time series between a voxel and its surrounding neighbors, typically including 26 adjacent voxels within a 3 × 3 × 3 cubic kernel. The ReHo values reflect the degree of local functional coherence, providing insights into regional neural activity. In this study, the analysis was conducted on preprocessed data without applying spatial smoothing, ensuring the preservation of fine-grained local signal patterns. The difference region between control and stress groups was shown by an independent-sample *t*-test with GRF correction (voxel *p* < 0.01; cluster *p* < 0.05).

### 2.7. Resting-State Functional Connectivity

Seed-based correlation analysis was conducted to measure interregional functional connectivity (FC) in the rsfMRI data. In this study, the seed regions were selected based on the results of VBM and ReHo analyses, which both identified abnormalities in the posterior DG region. Therefore, the posterior DG was used as the seed region for calculating whole-brain voxel-wise functional connectivity. The average time course within the region of interest (ROI) was extracted and used as the reference signal. Pearson’s correlation coefficient was then calculated to assess the connectivity between the ROI and all other voxels in the brain. Fisher’s z-transformation was applied to the correlation coefficients to convert them into z-scores for further statistical analysis. Differences in functional connectivity between the control and stress groups were identified using independent-sample *t*-test with GRF correction (voxel *p* < 0.05; cluster size > 50).

### 2.8. ROI-ROI Functional Connectivity

Significant regions of resting-state functional connectivity differences were extracted for ROI-ROI analysis. The DG seed matched the voxel-wise analysis, while EC and PAG ROIs were derived from regions with significant differences between groups. Fisher’s z-transformation converted correlation coefficients to z-scores for statistical comparison. Group differences were tested using independent-sample *t*-tests.

### 2.9. Brain Region Identification

In this study, the Paxinos and Watson rat brain atlas was used for brain region localization. The rat brain atlas was first converted into Nii format using a homemade program, and then spatially registered with a homemade SIGMA rat template using the ANTs toolkit (https://github.com/ANTsX/ANTs, accessed on 20 June 2021). Brain region locations were identified by overlaying the atlas and the registration map.

## 3. Results

### 3.1. Juvenile Stress Construction

**Figure 1:**
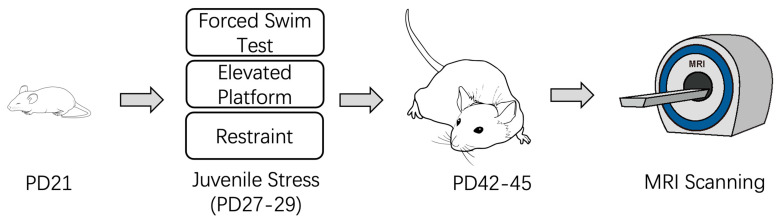
Experimental timeline of juvenile stress induction and MRI scanning.

This study employed a classic early-life stress model (Figure 1). Rats were weaned on postnatal day 21 (PD21) and randomly assigned to either the control group or the stress group. The stress group underwent short-term mild stress, including forced swimming, elevated platform exposure, and restraint, for three consecutive days from PD27 to PD29. After the stress protocol, all animals were returned to their cages for regular care. MRI scans were conducted on PD42-PD45 to evaluate the effects of stress. In our previous study, we performed behavioral tests to assess the effects of juvenile stress on adult rats’ social affiliation and social novelty preference [4,5].

In brief, the set-up for social affiliation and social novelty preference was a modified version of the three-chamber paradigm test [15]. The test arena did not have dividing walls, and two plastic containers with grids were placed at diagonal ends, used for housing the stranger rat or left empty. The subject animal was initially placed in the middle of the arena and allowed to freely explore. For social affiliation, the stranger rat was placed in one of the plastic containers, and the number and duration of direct contacts between the subject animal and the container housing the stranger rat were recorded for 10 min. For social novelty preference, a familiar rat from the same cage was placed in one container, and a novel stranger rat from a different cage was placed in the other container. The number of interactions and the latency of the first interaction delay with two containers were recorded for 10 min.

The results of social affiliation preference showed that there were no significant differences in the number of visits to the container with the stranger rat or the empty container in the stress group (stranger rat *p* = 0.4163; empty cup *p* = 0.0942). However, the stress group showed a significant reduction in the number of interactions with the stranger rat (*p* < 0.0001), indicating impaired sociability in these juvenile stressed rats. The results of social novelty preference showed that the stress group exhibited significantly higher latency to first contact with the stranger rat compared to the familiar rat (*p* = 0.0038), whereas the control group showed a preference for interacting with the stranger rat (*p* = 0.0086). This indicates that the stress group displayed a preference for interacting with the familiar rat, while the control group showed a greater preference for the stranger rat [4,5].

### 3.2. Brain Volume Changes

**Figure 2:**
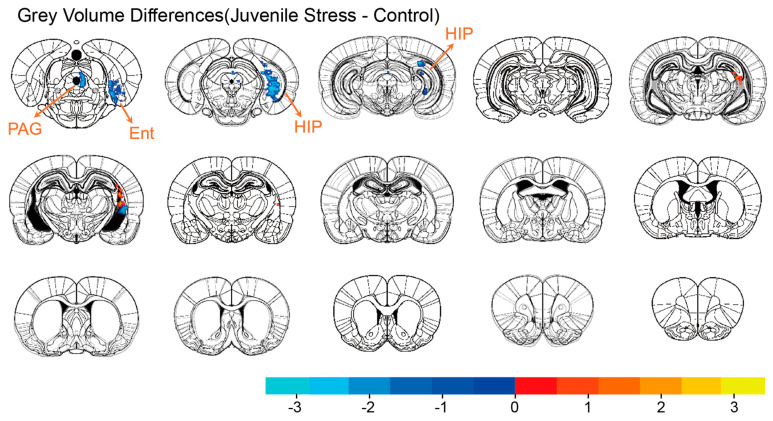
Significant differences were observed between the gray matter probability intensity maps of the juvenile stress group and the control group. A two−sample *t*−test was conducted for the comparison, and significant voxels were retained and displayed after GRF correction (voxel *p* < 0.05; cluster *p* < 0.05). Blue regions indicate reduced volume, while red regions show increased volume in the juvenile stress group compared to the control group.

We first compared the changes in brain gray matter volume between the stress and control groups (Figure 2). The results showed that several regions in the stress group had significantly smaller gray matter volume at PD42 compared to the control group. The regions with volume reductions included the PAG (peak *t* = −3.094; *p* = 0.004), Ent (peak *t* = −2.841; *p* = 0.008), and DG (peak *t* = −3.568; *p* = 0.001). To exclude the effects of body weight and overall brain volume, we also compared the animals’ body weight and total brain volume, and the results indicated that neither body weight nor total brain volume showed significant changes (Supplemental Materials, Figures S1 and S2).

### 3.3. Brain Regional Activity Changes

**Figure 3:**
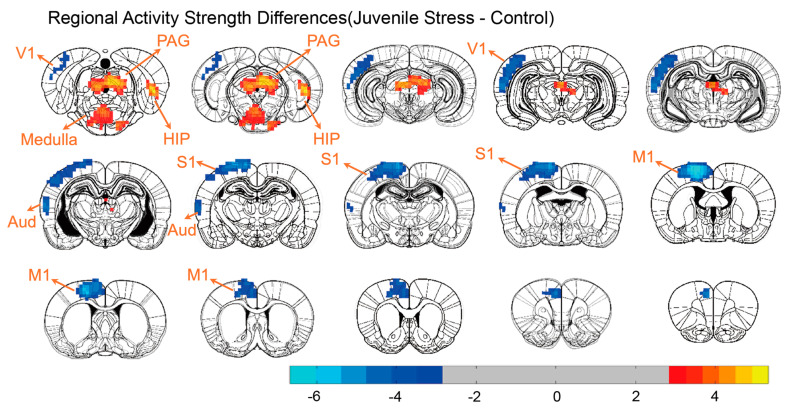
Significant differences were observed between the ReHo maps of the juvenile stress group and the control group. A two−sample *t*−test was performed for the comparison, and significant voxels were retained and displayed after GRF correction (voxel *p* < 0.01; cluster *p* < 0.05). Blue indicates areas with reduced ReHo in the juvenile stress group compared to the control group, while red highlights regions with increased ReHo in the juvenile stress group compared to the control group.

To compare the regional brain activity differences, we used ReHo to reflect local brain activity (Figure 3). The results of the independent two-sample *t*-test revealed a decrease in activity in cortical regions, primarily in the primary sensory areas (*t* = −6.658; *p* < 0.001), and an increase in activity in subcortical regions. The areas showing reduced activity in the cortical regions included the primary motor cortex, primary somatosensory cortex, primary auditory cortex, and primary visual cortex. In contrast, the regions with increased local activity were primarily concentrated around the PAG (*t* = 4.751; *p* < 0.001), the DG region of the hippocampus (*t* = 5.34; *p* < 0.001) and medulla (*t* = 3.903; *p* < 0.001).

### 3.4. Functional Connectivity Changes

**Figure 4:**
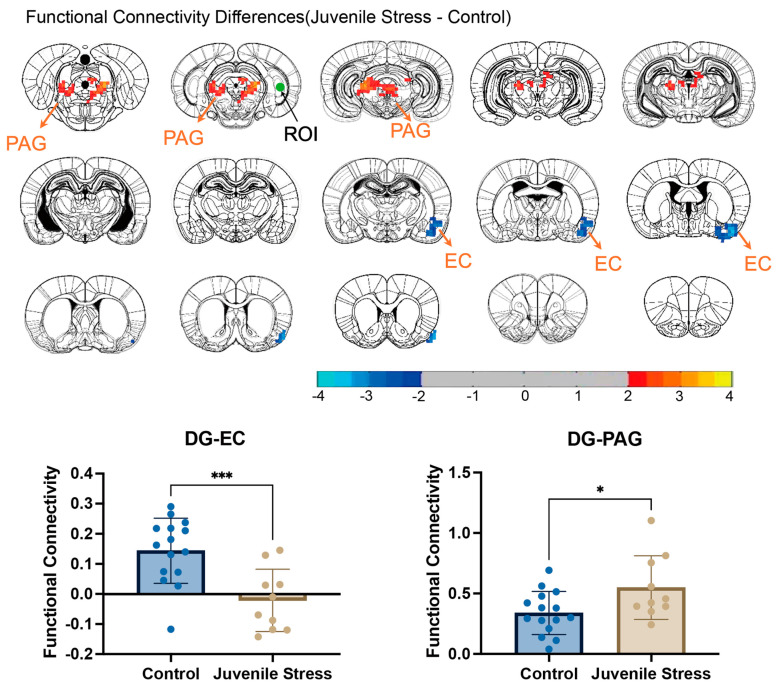
Functional connectivity (FC) differences between the juvenile stress and control groups. Significant voxels were identified using two−sample *t*−tests, with thresholding and multiple comparison correction applied (GRF correction). The DG seed is marked by green spheres. Blue regions indicate reduced FC, and red regions show increased FC in the juvenile stress group. ROI−based comparisons for DG−EC and DG−PAG connectivity were conducted using *t*−tests, with significance levels denoted as ‘*’ *p* < 0.05 and ‘***’ *p* < 0.001.

Since abnormalities were found in both brain structure and local function in the hippocampal DG region, and given that previous studies have primarily focused on the hippocampus and its association with social memory-related behaviors, we sought to further explore the changes in network structures associated with the hippocampus. The DG region occupies a significant portion of the subcortical areas in rats and has an irregular shape. To improve consistency within the seed point, we selected the posterior DG region as the seed point and calculated the functional connectivity strength between the posterior DG and all brain voxels. Comparison between the stress and control groups revealed that the functional connectivity strength between the DG and entorhinal cortex (EC) was significantly reduced in the stress group (Figure 4), with the mean value dropping sharply from 0.15 to approximately 0 (*t* = 3.801; *p* = 0.0009). In contrast, the functional connectivity strength between the DG and PAG significantly increased, with the mean value rising from 0.4 to around 0.5 (*t* = 2.386; *p* = 0.0256).

## 4. Discussion

This study explores the multidimensional impact of juvenile stress on the brain. Using functional magnetic resonance imaging (fMRI), we observed that juvenile stress not only affects the volume of the hippocampus (HIP) but also induces alterations in local brain activity and functional connectivity associated with the HIP. In addition to the structural and functional abnormalities of the HIP, the study also identified functional alterations in the periaqueductal gray (PAG), entorhinal cortex (Ent), and primary somatosensory cortex, as well as disrupted connectivity patterns in DG-EC and DG-PAG pathways. These findings provide preliminary evidence for structural and functional changes in the brain associated with stress-induced behavioral alterations.

This study found a significant reduction in gray matter volume in the DG, PAG, and Ent regions in the juvenile stress group at PD42. Extensive research has shown that juvenile stress can lead to adolescent social dysfunction, including reduced interest in novel objects, highlighting a link between early-life stress and memory function [4]. In early life, a critical period for neural development, synaptic connections are undergoing extensive formation and refinement, making this stage particularly susceptible to the detrimental effects of stress [16]. Exposure to stress during this time can disrupt neuronal connectivity development, potentially impair synaptic plasticity, and result in reductions in brain volume [17,18]. The DG region is primarily involved in the formation of new memories and the encoding of spatial information. It receives inputs from the entorhinal cortex and is responsible for pattern separation, which involves distinguishing between similar experiences or environments. The projections from the DG mainly target the CA3 region of the hippocampus, where memory consolidation occurs [19]. In a postmortem study, schizophrenia was associated with neuronal loss and other alterations in the hippocampus, particularly in area CA2. These changes include a reduction in the density of parvalbumin-expressing interneurons and impaired synaptic plasticity, which may contribute to cognitive and social dysfunction in affected individuals [12]. In addition, the PAG and Ent regions also appear to play critical roles in social memory. The PAG, which is involved in pain processing, threat detection, and emotional regulation, may experience structural changes due to juvenile stress. These alterations could disrupt stress responses and emotional regulation, aligning with previous findings that suggest the PAG’s involvement in modulating social behaviors and responses to stress [12]. The Ent region, serving as a vital hub connecting the hippocampus to the cortical areas, may reflect disrupted communication between these regions. This disruption could impair the integration of sensory and contextual information necessary for cognitive and emotional functions. Alterations in the Ent may therefore represent a mechanism underlying deficits in higher-order cognitive and social processes observed following juvenile stress [9].

From the perspective of brain activity, our results show a widespread activity suppression in cortical activity, particularly in the primary somatosensory, motor, auditory, and visual cortex. This decline in cortical activity may reflect broad cognitive impacts of juvenile stress, particularly interfering with basic functions such as attention, sensory input processing, and motor control. Social avoidance, commonly seen in anxiety and stress-related disorders, is often associated with the altered processing of social cues and reduced engagement with social environments [20]. In the case of juvenile stress, the suppression of cortical activity could impair the ability to appropriately perceive and respond to social stimuli, leading to avoidance behaviors. For example, compromised sensory processing (such as visual or auditory stimuli) may make social interactions more overwhelming or difficult to navigate, contributing to withdrawal from social situations. Additionally, deficits in motor control could lead to difficulties in social communication, further reinforcing the tendency toward social avoidance [21]. Thus, cortical activity suppression could provide a neural basis for the social dysfunction observed in juvenile stress [22].

In contrast, local activity in the hippocampal DG and PAG regions was significantly increased. The increased activity in the hippocampus may be related to altered memory processing and emotional regulation, suggesting that juvenile stress might enhance hippocampal activity to maintain certain functions. The increased activity in the PAG could be associated with heightened stress responses, as the PAG plays a critical role in threat processing and stress responses [23]. The discrepancy between the structural volume reduction and the increased local functional activity in the HIP and PAG may reflect stress-induced alterations in the development and function of GABAergic interneurons. Since GABAergic interneuron maturation is still ongoing during early life [24], juvenile stress may affect the development of these neurons. Stress during critical developmental periods has been shown to disrupt the maturation of GABAergic interneurons, leading to impaired inhibitory control and heightened neural excitability [25]. In the HIP, reduced gray matter volume could indicate compromised neurodevelopmental processes, while the observed increase in regional activity may result from the compensatory hyperactivity of the remaining excitatory circuits. Similarly, in the PAG, diminished inhibitory regulation might amplify local activity as an adaptive response to stress. Other than the HIP and PAG, enhanced local neuronal activity was also observed in the medulla region. The medulla plays a critical role in regulating breathing, heart rate, and autonomic nervous system activity [26]. Stress, whether emotional or physical, often leads to increased medullary activity as part of the body’s physiological response. This heightened activity may reflect the activation of the sympathetic nervous system in response to stress [27].

We observed unilateral effects in certain brain regions (e.g., PAG and DG), which may be attributed to natural hemispheric asymmetry in brain function. Since the stress paradigms we used—such as elevated plus maze, forced swim, and restraint stress—do not involve clear lateralized stimuli, we believe that this lateralization may result from differences in hemispheric sensitivity to the stressors. Specifically, the left and right hemispheres may exhibit different responses to the same stress, contributing to the observed unilateral activation [28]. Additionally, we acknowledge several limitations in this study, including the relatively small sample size and the exclusive use of male rats, which may impact the generalizability of the findings. These limitations will be addressed in future research to better understand the full scope of stress-related brain changes.

Functional connectivity analysis revealed a significant decrease in DG-EC connectivity and a significant increase in DG-PAG connectivity. The DG receives inputs from the EC via the perforant path and is crucial for pattern separation, a process essential for distinguishing similar experiences or memories [29]. This DG-EC interaction supports effective memory encoding and retrieval and the integration of sensory information with higher-order cognitive functions. A reduction in DG-EC connectivity, as observed in this study, can disrupt these processes, potentially impairing spatial memory and contextual learning [30]. These cognitive deficits are foundational to adaptive social behaviors, and disruptions in this connectivity may manifest as difficulties in interpreting social cues or navigating social contexts, aligning with behavioral phenotypes such as social avoidance and reduced novelty-seeking observed in juvenile stress models [4]. Previous studies have demonstrated reduced long-term potentiation (LTP) in HIP-EC pathways under stress, corroborating the vulnerability of this circuit to stress-induced alterations [4]. On the other hand, functional and structural abnormalities in PAG-HIP circuits have been reported in previous research. The PAG, a central region involved in emotional regulation and stress responses, exhibits increased connectivity with the DG, suggesting an adaptive but potentially maladaptive reorganization to cope with external stressors and threats. While direct evidence of PAG-HIP connectivity changes in response to juvenile stress is limited, studies indicate that stress broadly impacts midbrain–HIP circuits [31,32]. For example, the PAG’s role in modulating fear memory aligns with findings of increased PAG-DG connectivity, potentially reflecting compensatory alterations in emotional regulation pathways under stress [33]. These multidimensional connectivity changes highlight how juvenile stress reconfigures hippocampal and midbrain circuits, contributing to both cognitive and emotional dysregulation [34].

Although our results have revealed abnormalities in brain regions with structural and functional MRI, this influence of juvenile stress on rats urgently requires further experimental confirmation. Additional fundamental experiments would be conducted in future to corroborate the MRI findings. (1) Microstructural Validation: Employing histopathological methods (e.g., Nissl staining and electron microscopy) to quantify neuronal density and synaptic ultrastructure, establishing biological correlations between microscopic alterations and macroscopic neuroimaging features. (2) Electrophysiological Mechanisms: Combining in vivo electrophysiological recordings (local field potentials and multichannel arrays) with optogenetic interventions to decode the neurophysiological basis of functional connectivity changes. (3) Longitudinal Dynamics: Enhancing the reliability of the results by repeatedly assessing structural and functional changes in the rat brain at different developmental time points. 

## 5. Conclusions

In conclusion, this study provides macro-level evidence of the impact of juvenile stress on the brain through structural and functional MRI. The findings reveal that juvenile stress induces long-lasting behavioral effects on both brain structure and function. Notably, the hippocampus (HIP) and periaqueductal gray (PAG) regions play critical roles in the brain’s response to juvenile stress. This research is expected to offer valuable macro-level guidance for subsequent mechanistic studies.

## Data Availability

The original contributions presented in this study are included in the article/Supplementary Materials. Further inquiries can be directed to the corresponding authors.

## Supplementary Materials

The following supporting information can be downloaded at: https://www.mdpi.com/article/10.3390/brainsci15030284/s1, Figure S1: Weight Comparison in PD42; Figure S2: Whole brain volume comparison (Grey Matter + White Matter + CSF, olfactory bulb and cerebellum were excluded); Figure S3: Main regions with significant differences shown in results.

## References

[1] Romeo R.D., McEwen B.S. (2006). Stress and the adolescent brain. Ann. N. Y. Acad. Sci..

[2] Targum S.D., Nemeroff C.B. (2019). The Effect of Early Life Stress on Adult Psychiatric Disorders. Innov. Clin. Neurosci..

[3] Avital A., Richter-Levin G. (2005). Exposure to juvenile stress exacerbates the behavioural consequences of exposure to stress in the adult rat. Int. J. Neuropsychopharmacol..

[4] Raghuraman R., Navakkode S., Sajikumar S. (2023). Alteration of hippocampal CA2 plasticity and social memory in adult rats impacted by juvenile stress. Hippocampus.

[5] Raghuraman R., Manakkadan A., Richter-Levin G., Sajikumar S. (2022). Inhibitory metaplasticity in juvenile stressed rats restores associative memory in adulthood by regulating epigenetic complex G9a/GLP. Int. J. Neuropsychopharmacol..

[6] Sandi C., Haller J. (2015). Stress and the social brain: Behavioural effects and neurobiological mechanisms. Nat. Rev. Neurosci..

[7] Hitti F.L., Siegelbaum S.A. (2014). The hippocampal CA2 region is essential for social memory. Nature.

[8] Meira T., Leroy F., Buss E.W., Oliva A., Park J., Siegelbaum S.A. (2018). A hippocampal circuit linking dorsal CA2 to ventral CA1 critical for social memory dynamics. Nat. Commun..

[9] Raam T., McAvoy K.M., Besnard A., Veenema A.H., Sahay A. (2017). Hippocampal oxytocin receptors are necessary for discrimination of social stimuli. Nat. Commun..

[10] Tonegawa S., Pignatelli M., Roy D.S., Ryan T.J. (2015). Memory engram storage and retrieval. Curr. Opin. Neurobiol..

[11] Okuyama T., Kitamura T., Roy D.S., Itohara S., Tonegawa S. (2016). Ventral CA1 neurons store social memory. Science.

[12] Piskorowski R.A., Nasrallah K., Diamantopoulou A., Mukai J., Hassan S.I., Siegelbaum S.A., Gogos J.A., Chevaleyre V. (2016). Age-Dependent Specific Changes in Area CA2 of the Hippocampus and Social Memory Deficit in a Mouse Model of the 22q11.2 Deletion Syndrome. Neuron.

[13] Maggio N., Segal M. (2011). Persistent changes in ability to express long-term potentiation/depression in the rat hippocampus after juvenile/adult stress. Biol. Psychiatry.

[14] Lammertink F., van den Heuvel M.P., Hermans E.J., Dudink J., Tataranno M.L., Benders M.J., Vinkers C.H. (2022). Early-life stress exposure and large-scale covariance brain networks in extremely preterm-born infants. Transl. Psychiatry.

[15] Kaidanovich-Beilin O., Lipina T., Vukobradovic I., Roder J., Woodgett J.R. (2011). Assessment of social interaction behaviors. J. Vis. Exp..

[16] Kirmse K., Zhang C. (2022). Principles of GABAergic signaling in developing cortical network dynamics. Cell Rep..

[17] Kim J.J., Diamond D.M. (2002). The stressed hippocampus, synaptic plasticity and lost memories. Nat. Rev. Neurosci..

[18] Borsini A., Giacobbe J., Mandal G., Boldrini M. (2023). Acute and long-term effects of adolescence stress exposure on rodent adult hippocampal neurogenesis, cognition, and behaviour. Mol. Psychiatry.

[19] Leutgeb J.K., Leutgeb S., Moser M.B., Moser E.I. (2007). Pattern separation in the dentate gyrus and CA3 of the hippocampus. Science.

[20] Fox A.S., Kalin N.H. (2014). A translational neuroscience approach to understanding the development of social anxiety disorder and its pathophysiology. Am. J. Psychiatry.

[21] LeDoux J.E. (2000). Cognitive-emotional interactions: Listen to the brain. Cogn. Neurosci. Emot..

[22] Dopfel D., Zhang N. (2018). Mapping stress networks using functional magnetic resonance imaging in awake animals. Neurobiol. Stress.

[23] Motta S.C., Carobrez A.P., Canteras N.S. (2017). The periaqueductal gray and primal emotional processing critical to influence complex defensive responses, fear learning and reward seeking. Neurosci. Biobehav. Rev..

[24] Kilb W. (2012). Development of the GABAergic system from birth to adolescence. Neuroscientist.

[25] Pan N.C., Fang A., Shen C., Sun L., Wu Q., Wang X. (2019). Early excitatory activity-dependent maturation of somatostatin interneurons in cortical layer 2/3 of mice. Cereb. Cortex.

[26] Colombari E., Sato M.A., Cravo S.L., Bergamaschi C.T., Campos R.R., Lopes O.U. (2001). Role of the Medulla Oblongata in Hypertension. Hypertension.

[27] Li J., Yang R., Xia K., Wang T., Nie B., Gao K., Chen J., Zhao H., Li Y., Wang W. (2018). Effects of stress on behavior and resting-state fMRI in rats and evaluation of Telmisartan therapy in a stress-induced depression model. BMC Psychiatry.

[28] Meyer M.A.A., Anstötz M., Ren L.Y., Fiske M.P., Guedea A.L., Grayson V.S., Schroth S.L., Cicvaric A., Nishimori K., Maccaferri G. (2020). Stress-related memories disrupt sociability and associated patterning of hippocampal activity: A role of hilar oxytocin receptor-positive interneurons. Transl. Psychiatry.

[29] Schmidt B., Marrone D.F., Markus E.J. (2012). Disambiguating the similar: The dentate gyrus and pattern separation. Behav. Brain Res..

[30] Schapiro A.C., Turk-Browne N.B., Botvinick M.M., Norman K.A. (2017). Complementary learning systems within the hippocampus: A neural network modelling approach to reconciling episodic memory with statistical learning. Philos. Trans. R. Soc. B Biol. Sci..

[31] Xiao Q., Zhou X., Wei P., Xie L., Han Y., Wang J., Cai A., Xu F., Tu J., Wang L. (2021). A new GABAergic somatostatin projection from the BNST onto accumbal parvalbumin neurons controls anxiety. Mol. Psychiatry.

[32] Chen A.P.F., Chen L., Kim T.A., Xiong Q. (2021). Integrating the Roles of Midbrain Dopamine Circuits in Behavior and Neuropsychiatric Disease. Biomedicines.

[33] McNaughton B.L., Battaglia F.P., Jensen O., Moser E.I., Moser M.-B. (2006). Path integration and the neural basis of the ‘cognitive map’. Nat. Rev. Neurosci..

[34] Johnson F.K., Delpech J.-C., Thompson G.J., Wei L., Hao J., Herman P., Hyder F., Kaffman A. (2018). Amygdala hyper-connectivity in a mouse model of unpredictable early life stress. Transl. Psychiatry.

