# Supplementary material for: Variations of Aberrant Volume, Activity, and Network Connectivity of Hippocampus in Adolescent Male Rats Exposed to Juvenile Stress
Source: Brain Sci. 2025 Mar 7;15(3):284. doi: 10.3390/brainsci15030284 (PMC11940772; doi:10.3390/brainsci15030284)
Supplement: Supplementary file 1 [file brainsci-15-00284-s001.zip › brainsci-3486042-supplementary.pdf]

Supplemental Materials

Weight of Animals

No significant differences were observed in the weight between the control group (n = 15) and the juvenile stress group (n = 11) at PD42.

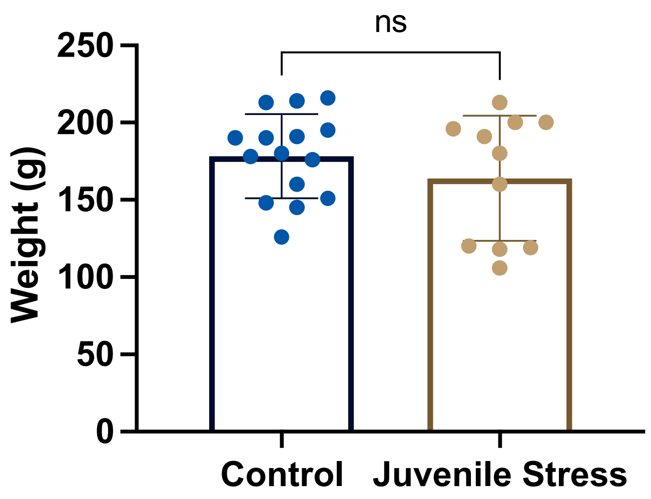

Figure S1. Weight Comparison in PD42. Two-sample *t*-test was used to evaluate the differences between control group and juvenile stress group ("ns" represented no significant difference).

Whole brain Volume of Animals

No significant differences were observed in the whole brain volume (Grey Matter + White Matter + CSF, olfactory bulb and cerebellum were excluded) between control group (n = 15) and juvenile stress group (n = 11) at PD42 days.

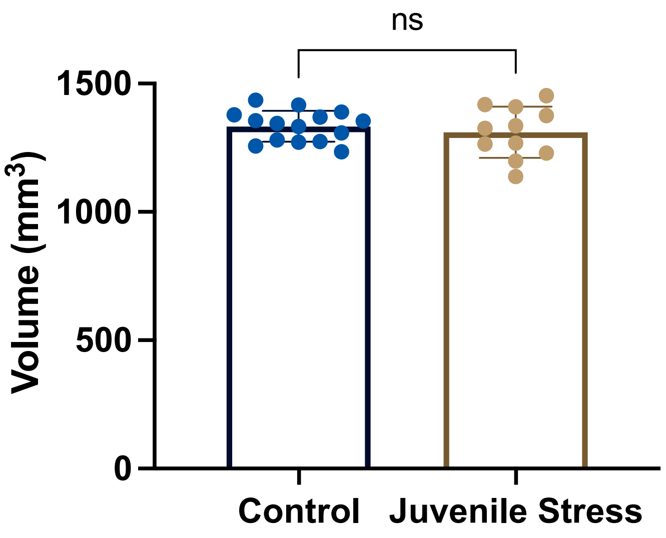

Figure S2. Whole brain volume comparison (Grey Matter + White Matter + CSF, olfactory bulb and cerebellum were excluded). Two-sample *t*-test was used to evaluate the differences between control group and juvenile stress group ("ns" represented no significant difference).

Definition of brain regions

The main brain regions with significant differences identified in this study include S1, M1, PAG and HIP.

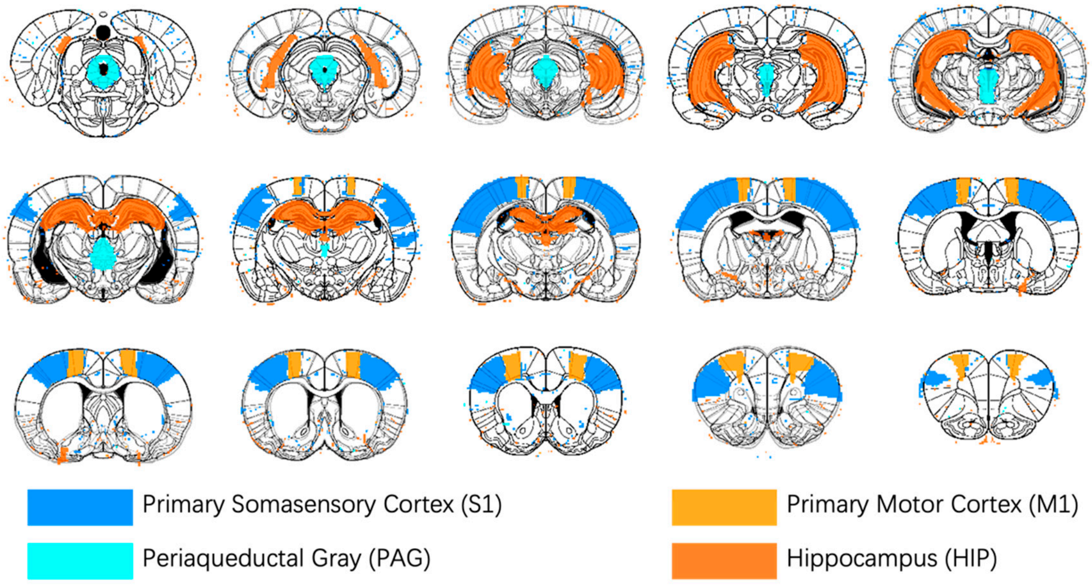

Figure S3. Main regions with significant differences shown in results.
